# Remarkable Thermal Conductivity of Epoxy Composites Filled with Boron Nitride and Cured under Pressure

**DOI:** 10.3390/polym13060955

**Published:** 2021-03-20

**Authors:** Sasan Moradi, Frida Román, Yolanda Calventus, John M. Hutchinson

**Affiliations:** Departament de Màquines i Motors Tèrmics, ESEIAAT, Universitat Politècnica de Catalunya, C/Colom 11, 08222 Terrassa, Spain; sasan.moradi@upc.edu (S.M.); frida.roman@upc.edu (F.R.); yolanda.calventus@upc.edu (Y.C.)

**Keywords:** thermal conductivity, epoxy composites, boron nitride, density, pressure, differential scanning calorimetry (DSC)

## Abstract

This work demonstrates that the application of even moderate pressures during cure can result in a remarkable enhancement of the thermal conductivity of composites of epoxy and boron nitride (BN). Two systems have been used: epoxy-thiol and epoxy–diamine composites, filled with BN particles of different sizes and types: 2, 30 and 180 μm platelets and 120 μm agglomerates. Using measurements of density and thermal conductivity, samples cured under pressures of 175 kPa and 2 MPa are compared with the same compositions cured at ambient pressure. The thermal conductivity increases for all samples cured under pressure, but the mechanism responsible depends on the composite system: For epoxy–diamine composites, the increase results principally from a reduction in the void content; for the epoxy–thiol system with BN platelets, the increase results from an improved matrix-particle interface; for the epoxy–thiol system with BN agglomerates, which has a thermal conductivity greater than 10 W/mK at 44.7 vol.% filler content, the agglomerates are deformed to give a significantly increased area of contact. These results indicate that curing under pressure is an effective means of achieving high conductivity in epoxy-BN composites.

## 1. Introduction

Epoxy composites filled with boron nitride (BN) are widely used for heat management in Insulated Metal Substrates (IMS). Such materials must satisfy a number of requirements, including adhesion, processability, electrical insulation, and high thermal conductivity. The last of these has attracted considerable attention, and the state of the art in polymer-based composites in general [1,2], and in epoxy-BN composites in particular [3], has been reviewed recently. There are numerous parameters which play a role in determining the thermal conductivity, perhaps the most important being the filler content, size and type (shape) of filler particles, and the surface treatment or the use of coupling agents.

It is widely recognized that the thermal conductivity of epoxy-BN systems increases with increasing BN content [3,4], at least up to about 50 vol.% BN. However, higher BN contents present difficulties for processing on account of their being very stiff pastes, and the thermal conductivity often decreases as a consequence of the presence of voids which are difficult to remove. The effects of BN filler size and type have been investigated previously [5,6], and it has been shown that, for a given filler content, the thermal conductivity generally increases with particle size; the usual interpretation is that larger particles have a smaller interfacial contact area with the matrix, this interface presenting a barrier to phonon transport. As regards filler type, it was found [6] that agglomerates result in a higher thermal conductivity than do platelets.

The effect of BN filler size highlights the importance of the matrix–particle interface, and attempts have been made to improve this interface by the surface treatment of the particles or with the use of coupling agents. The results, however, are not entirely conclusive. While many authors report significant increases in the thermal conductivity [7,8,9,10,11,12,13,14] there are also reports that these treatments have much less effect [15,16,17].

The drive towards increasing the thermal conductivity of epoxy composites for IMS applications, occasioned by the demand for the use of electronic devices at ever higher frequencies and power densities, should take into consideration all of the above aspects, but must also be governed by some more practical motives. For example, the electrical insulation properties can be affected by the migration of copper ions in the formation of conductive anodic filaments in humid conditions [18,19], which can mitigate against the use of amines as a cross-linking agent. Similarly, the requirement for good adhesion of the dielectric layer to the metallic substrate of the IMS would imply a preference for filler particles such as Al_2_O_3_ rather than BN, despite the intrinsically lower thermal conductivity of the former. Processability is also a factor that must be borne in mind; although significantly higher thermal conductivities have been achieved in epoxy composites by the alignment of the filler particles by various means (e.g., [20]), these procedures are usually rather elaborate, and hence the composites are not easily fabricated.

In the light of the above discussion, it would be interesting to find a more generic way in which the thermal conductivity of epoxy composites might be increased. There is, indeed, just such a possibility, though it has received little attention to date; this is the effect of the application of pressure during cure. In the first place, it might intuitively be expected that applying pressure would consolidate the material, improving the matrix–filler interface, and hence enhancing the thermal conductivity. Additionally, though, there is also the possibility of densifying the material, by cooling the epoxy matrix through its glass transition region under pressure [21,22]. In view of the occasionally noted correlation between density and thermal conductivity [22,23], this would imply a further possible route towards increasing the thermal conductivity. Accordingly, it would be appropriate to summarize here the state of the art as regards the use of pressure during the cure of epoxy-BN composites for high thermal conductivity applications.

Several authors have reported the use of pressure during fabrication of their epoxy-BN composites, but there are very few systematic studies of this effect on the thermal conductivity. Furthermore, there are several different ways in which the pressure can be applied. Some workers apply pressure to the composite mixture before curing at ambient pressure [23,24,25]. The principal objective is presumably to consolidate the sample, though a wide range of pressures is used, and other objectives are noted: Lewis et al. [25] use approximately 1.6 MPa to “break … agglomerations and flatten the material”; Isarn et al. [24] use approximately 74 MPa to “compact and shape” cylindrical samples before cure, while Zhu et al. [23] compression mold samples, at pressures in the range from 43 to 215 MPa, in such a way as to introduce significant orientation, as evidenced by a marked difference between the thermal conductivities measured in the in-plane and through-plane directions.

On the other hand, several workers simply use pressure, without specifying the magnitude, in a process often referred to as “hot pressing”, as part of the fabrication procedure of their epoxy composites [13,26,27,28,29]. The primary objectives of each of these studies are manifold, and do not necessarily include the investigation of the effect of pressure. For example, Jang et al. [13] and He et al. [26] study the effect of coupling agents and functionalization of the BN particles on the thermal conductivity of their hot-pressed composites, while Mun et al. [28] and Wu et al. [29] report the thermal conductivity of hybrid epoxy-BN composites with expanded graphite and silver nanoparticles, respectively, in both cases after compression molding at an unspecified pressure. In contrast, Sun et al. [27] use the hot pressing process to deliberately introduce orientation into their cured composites fabricated with BN platelets, in comparison with composites fabricated with BN microspheres, which are essentially isotropic. Thus, although these composites are all cured under pressure, the effect of pressure *per se* on the thermal conductivity cannot be identified.

In the majority of studies, though, the composite is either first partially cured under pressure [8,12,30,31,32,33] and subsequently post-cured without pressure, or is directly fully cured [7,34,35,36], in all cases under specified conditions of pressure, temperature and cure time. Distinction should be made here between two different modes in which the pressure is applied: (i) the pressure is applied uniaxially (hot-pressing) [31,32,33], which deliberately introduces orientation and yields composites with significant differences between the in-plane and out-of-plane thermal conductivities; and (ii) the sample is compressed in a mold [8,12,30,34,35,36], thus implying effectively hydrostatic pressure and no orientation of the BN filler particles. A variety of pressures is employed in the fabrication of the composites, but for the hot-pressing technique [31,32,33] the pressures tend to be lower, in the range from 200 kPa (30 psi) to 13 MPa, in comparison with pressures of 5 MPa [12], 10.5 MPa [7], 13.2 MPa [36], 20.6 MPa (3000 psi) [8,30], and 42 MPa [34,35] for samples cured in a mold.

In the latter category, the large number of additional factors which play a role in determining the thermal conductivity of these composites (e.g., BN particle size and shape, filler content, surface treatments and coupling agents [3]) make it impossible to identify the effect of pressure. For example, the increasing pressures listed immediately above give rise to the following maximum thermal conductivities: 2.7 W/mK and 10.6 W/mK for 60 vol.% of surface treated 3.6 μm and 10.6 μm platelets, respectively, cured at 5 MPa [12]; 10.31 W/mK for 57 vol.% of 5–11 μm silane treated agglomerates, cured at 10.5 MPa [7]; 3.6 W/mK for 69 vol.% of untreated BN particles, cured at 13.2 MPa [36]; 1.5 W/mK and 3.5 W/mK for 80 vol.% of surface treated 6 μm and 18 μm particles, respectively, cured at 20.6 MPa [30]; 3.4 W/mK for 37 vol.% of surface treated BN platelets, cured at 42 MPa [34].

In order to identify the effect of pressure on the thermal conductivity of these epoxy-BN composites, it would clearly be necessary to make a systematic study. However, of all the studies discussed above, only Zhu et al. [23] and Hu et al. [33] include such an investigation. Zhu et al. [23], as noted earlier, used pressures between 43 and 215 MPa to introduce significant orientation to the uncured epoxy-BN mixture at room temperature before curing without pressure at higher temperature. For example, these authors reported a large increase in the thermal conductivity, reaching ~20 W/mK for 90 wt.% BN in the in-plane (oriented) direction in comparison with about 10 W/mK in the through-plane direction and with only about 4 W/mK for samples with the same filler content prepared without pressure. There was no significant effect on the thermal conductivity of the magnitude of the pressure applied, which suggests that 43 MPa pressure is sufficient to introduce significant orientation, which is not further improved by higher pressures. In contrast, Hu et al. [33] used lower pressures and a much more elaborate fabrication procedure. Samples were prepared by first pre-curing the epoxy-BN mixture to increase its viscosity, then hot-pressing at different pressures (7, 10, 13 MPa) and temperatures (130, 150, 170 °C) before shaping the samples in a cylindrical mold and finally curing them without pressure at 120 °C for 1 h, 160 °C for 2 h, and 200 °C for 2 h. These authors found that the thermal conductivity increased with pressure, for example from 8.3 W/mK to 10.9 W/mK and then to 11.9 W/mK for 60 wt.% of 18 μm BN particles at 7, 10, and 13 MPa. Thus, at these pressures, significantly lower than those used by Zhu et al. [23], the degree of orientation *is* a function of the pressure applied.

From the foregoing discussion, it is evident that it is not possible to obtain any clear idea about the effect of pressure during cure on the thermal conductivity of epoxy-BN composites from the results presented in the literature. Consequently, in the present work we make a systematic investigation of the effect of pressure during cure on the thermal conductivity of a number of different epoxy-BN composite systems, and show that significant increases can be achieved. The mechanisms by which these increases are obtained depend on the composite system, and demonstrate that enhancement of the thermal conductivity can occur in ways other than by orientation.

## 2. Materials and Methods

### 2.1. Materials

The epoxy resin used was diglycidyl ether of bisphenol-A, DGEBA (Araldite GY240, Huntsman Advanced Materials, Salt Lake City, UT, USA), with a nominal molecular weight per epoxy equivalent (eq) of 182 g/eq, density of 1.17 g/cm^3^ and viscosity of 7000 to 9000 mPa.s at 25 °C. The principal cross-linking agent used was a thiol, pentaerythritol tetrakis (3-mercaptopropionate) (Sigma-Aldrich, Saint Louis, MO, USA), with a molecular weight of 488.66 g/mol, density of 1.28 g/cm^3^, and viscosity of 500 mPa.s at 23 °C. In order to initiate the cross-linking reaction of the epoxy with the thiol, a latent initiator, encapsulated imidazole (LC-80, Technicure, A&C Catalysts, Linden, NJ, USA) in the form of powder, was used. In addition, a polyoxypropylene diamine, Jeffamine D-230 (molecular weight 230 g/mol, density 0.948 g/cm^3^, viscosity 9000 mPa.s at 25 °C), and a dicyandiamide, N,N-dimethyl-N-phenyl urea (PDU-250M, Technicure, A&C Catalysts, Linden, NJ, USA), were also used as alternative cross-linking agent and initiator, respectively, for comparative purposes.

**Figure 1:**
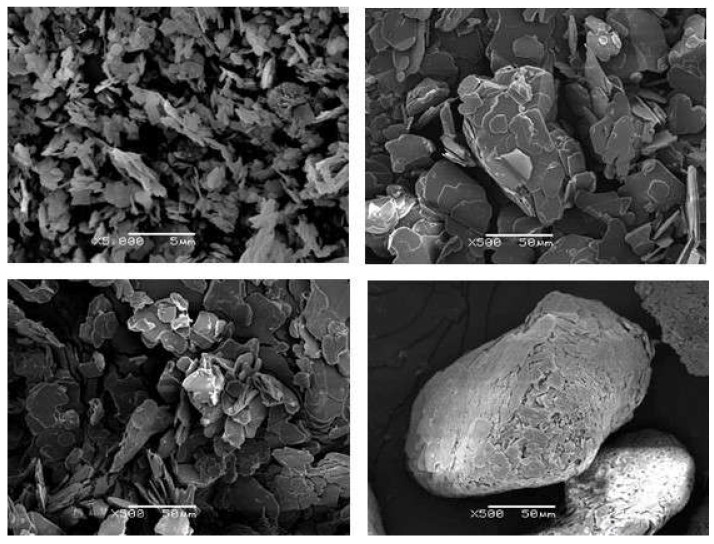
Scanning Electron Microscopy (SEM) images of the as-received BN particles: (top left) PCTP2, 2 μm, magnification 5000×, scale bar 5 μm; (top right) PCTP30, 30 μm, magnification 500×, scale bar 50 μm; (bottom left) PCTP30D, 180 μm, magnification 500×, scale bar 50 μm; (bottom right) CTS7M, 120 μm, magnification 500×, scale bar 50 μm.

Different grades of hexagonal BN filler (Saint Gobain Ceramic Materials, Amherst, NY, USA) were used, shown in Figure 1, with average and maximum particle size, tap density and specific surface area, respectively, as given in the manufacturer’s literature [37], as follows:

- PCTP2: 2 μm, 10 μm, 0.2 g/cm^3^, 10 m^2^/g;
- PCTP30: 30 μm, 100 μm, 0.6 g/cm^3^, 1 m^2^/g;
- PCTP30D: 180 μm, 1600 μm, 0.6 g/cm^3^, 1 m^2^/g;
- CTS7M: 120 μm, 180 μm, 0.5 g/cm^3^, 3.5 m^2^/g.

Various important aspects of these filler particles can be appreciated from this Figure. The 2 μm and 30 μm particles are both in the form of platelets, but the specific surface area is 10 times higher for the smaller particles; this has the effect that it is difficult to fabricate composites with a large content of the 2 μm particles. The 180 μm particles are also in the form of platelets, but there is a very wide distribution of particle sizes; according to the manufacturer, these particles are engineered for high shear mixing processes. On the other hand, the 120 μm particles are in the form of agglomerates, denoted as spherical powder by the manufacturer. The filler particles were used as received, without any surface treatment.

### 2.2. Methods

#### 2.2.1. Sample Preparation

The epoxy-BN composites were fabricated in the proportions given in Table 1, where the sample nomenclature is also given. In all cases, a stoichiometric ratio of epoxy to curing agent was used, and for the epoxy–thiol composites the initiator was added in a proportion of 2 parts per hundred resin. For each epoxy system cured under pressure (except for the 2 μm composites, for which only the lower filler content was used, for the reasons given above), two BN filler loadings were used, specified with respect to the combined weight of epoxy and BN: For epoxy–thiol, 60% and 70%; for epoxy–diamine, 60% and 65%. The reason why a lower maximum filler content was used for the epoxy–diamine composites lies in the significantly higher viscosity of the diamine (9000 mPa.s) in comparison with the thiol (500 mPa.s); epoxy–diamine composites with a filler content higher than 65% by weight of BN with respect to the combined weight of BN and epoxy resulted in a paste that was so stiff that it was unworkable in practice. Likewise, no 70% epoxy–thiol samples could be prepared at ambient pressure with the 120 μm agglomerates, though this was possible when pressure was applied during cure. This ability to obtain epoxy-BN composite samples with higher filler contents by the application of pressure was earlier noted by Hu et al. [33]. Table 1 also includes data for the 30% and 50% epoxy–thiol systems, which were prepared at ambient pressure.

**Figure 2:**
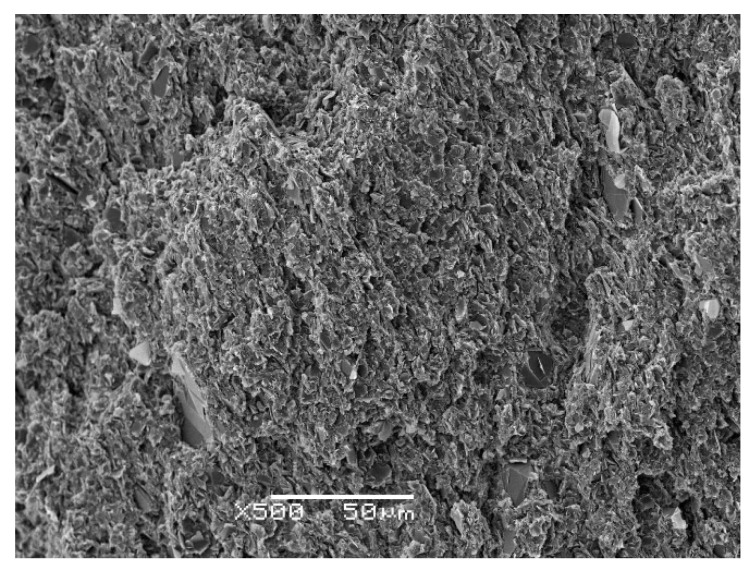
Scanning electron microscopy (SEM) image of the fracture surface of sample ETLBN2-60, magnification 500×, scale bar 50 μm.

All the components were mixed by hand for 15 min to ensure homogeneity and were then degassed under vacuum (<26 hPa) at room temperature for about 10 min. The quality of the dispersion can be evidenced by examination by Scanning Electron Microscopy (SEM) of the fracture surfaces of cured composite samples. An illustration of one such SEM micrograph is shown in Figure 2, for the sample ETLBN2-60, with 60% of 2 μm platelets. In comparison with composites fabricated with the other BN particles, these 2 μm particles present the greatest difficulty for dispersion in view of their high specific surface area. Nevertheless, it can be seen from Figure 2 that the dispersion is excellent; the particles appear to be uniformly distributed across the fracture surface, and there are no agglomerations of particles or regions rich in epoxy matrix. Similar SEM micrographs for other samples shown later demonstrate that this quality of dispersion is maintained for other compositions.

#### 2.2.2. Thermal Conductivity

The thermal conductivity was measured using the Transient Hot Bridge method (Linseis THB-100, Selb, Germany), in which a heat pulse is applied to a sensor placed between two surfaces of the sample material and the instrument measures the temperature change Δ*T* as a function of time, *t*, following the heat pulse input. The thermal conductivity, λ, was obtained [38] from an extrapolation of the linear part of a plot of Δ*T* as a function of the inverse square root of time, 1/√*t*. The instrument was calibrated with five different standards covering the range from 0.2 to 10 W/mK. The samples prepared at ambient pressure were cast in silicone molds 10 mm × 40 mm × 4 mm, and cured isothermally in an air-circulating oven at 70 °C for 3 h. Four measurements were made of the thermal conductivity of each of the samples, and the average value was taken. Typically, the standard deviation of these four measurements was ±0.1 W/mK.

#### 2.2.3. Compressed Samples

**Figure 3:**
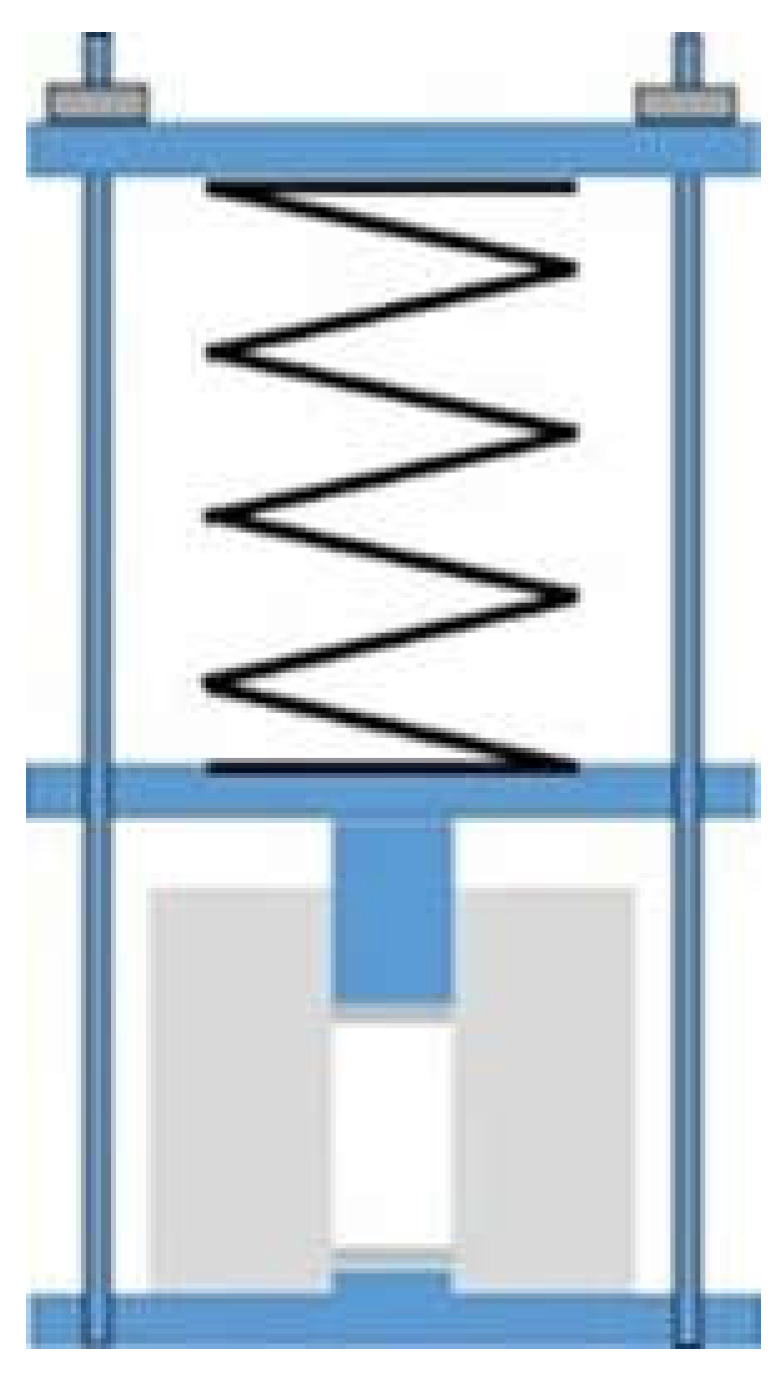
Schematic illustration of the compression device.

In order to obtain compressed samples, the device illustrated in Figure 3 was used. The required amount of epoxy–thiol or epoxy–diamine mixture was introduced into the Teflon cylinder of internal diameter 15 mm, outside diameter 60 mm and height 52 mm, and the spring (with spring constant 5.95 kN/m) was compressed by a measured distance and then locked in place. The force on the piston could be calibrated to give pressures up to 3 MPa, though results are presented here only for a maximum pressure of 2 MPa. The whole assembly was placed in an air-circulating oven such that the temperature of the sample remained at 70 °C for 3 h in order to effect the cure under the applied pressure. After cure, the pressure was maintained during cooling, and the cured sample was removed at room temperature. The cured samples, in the form of solid cylinders 15 mm diameter and between 25 and 35 mm in length, were cut in half using a diamond wafering saw to give two smooth and flat surfaces for the measurement of thermal conductivity. In an earlier arrangement, a weight was used in place of the spring, giving rise to a much smaller pressure of 175 kPa; results obtained with this much lower pressure are also included in the present work.

#### 2.2.4. Density

The density of the cured samples was measured by Archimedes method. The samples were first weighed in air at room temperature, and then when immersed fully in ethanol, suspended by a fine thread. From repeated measurements on the same sample, the uncertainty in the density is estimated as ±0.02 g/cm^3^.

#### 2.2.5. Scanning Electron Microscopy (SEM)

Fully cured samples, similar to those used for the thermal conductivity measurements and prepared using the same isothermal curing procedure, were fractured and then the fracture surface was examined in a Scanning Electron Microscope (JEOL JSM-5610, Tokyo, Japan). An accelerating voltage of 10 kV was used to give magnifications from 100× to 5000×.

## 3. Results and Discussion

### 3.1. Density Measurements

**Figure 4:**
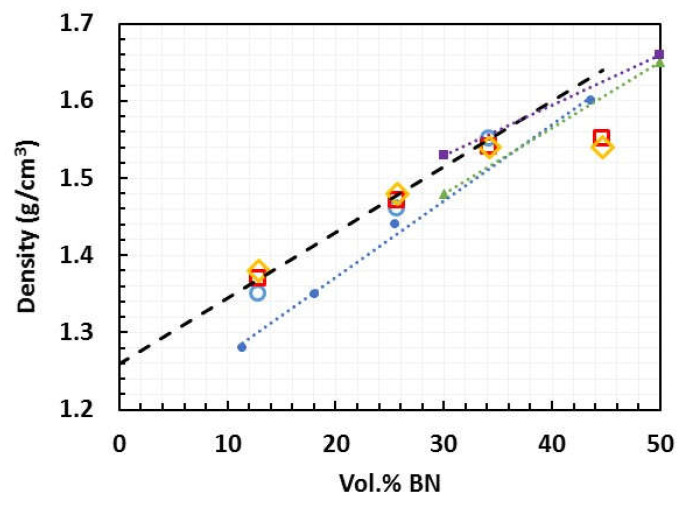
Density as a function of BN content for epoxy–thiol composites prepared at ambient pressure with different BN platelet sizes: 2 μm, open blue circles; 30 μm, open red squares; 180 μm, open yellow diamonds [5]. Filled symbols for data taken from the literature: blue circles, Lewis et al. [25]; green triangles, Chung and Lin, 3.6 μm [12]; purple squares, Chung and Lin, 10.6 μm [12].

The density of epoxy–thiol-BN platelet composites, samples ETLBN*x*-30, -50, -60, and -70, with *x* = 2, 30, and 180, cured in open molds without the application of pressure, was measured experimentally in earlier work [5], and is shown as a function of the vol.% of BN by the open symbols in Figure 4.

Here it can be seen that, for BN contents less than 40 vol.%, the density of the composites is independent of the BN particle size, and increases linearly with increasing filler content from a value of 1.25 g/cm^3^ for the epoxy–thiol system without any filler. In fact, the composite density calculated from the densities and weight fractions of the three components (epoxy, thiol, BN) and allowing for a 4% shrinkage on cure gives the dashed line, which agrees very closely with the experimental values, implying that there is an insignificant void content for filler volume fractions less than 34%. For the highest filler content of almost 45 vol.%, however, there is a significant deviation from this linear relationship, which is attributed to the presence of voids in the sample; at such high filler contents the composite is a very stiff paste, and the mixing procedure introduces air bubbles which cannot be removed by degassing.

Comparison can be made with literature values for other epoxy-BN composites, though this is not straightforward: the density depends on the nature of the epoxy and hardener system as well as on the BN content, and there are also rather few studies in which density measurements are reported. For epoxy-BN composites prepared by curing at ambient pressure, data are available from two references in addition to Zhu et al. [23]. Lewis et al. [25] use a DGEBA epoxy (1.17 g/cm^3^) cured with triethylenetetramine (0.982 g/cm^3^) and filled with 3–8 μm BN platelets (2.29 g/cm^3^), which gives a calculated density of the composite (without any allowance for shrinkage on cure) which falls on the same dashed line as given in Figure 4. The data of Lewis et al. are included in Figure 4 as the filled blue circles, and fall below the calculated line, as well as below our experimental values except for the highest BN content for which we noted above the presence of voids. The deviation of the data of Lewis et al. from the calculated line may be a result of their rather high value for the density of the BN particles, taken from the manufacturer’s literature [39]. If the value of 2.1 g/cm^3^ used in our calculations is applied instead, together with a 4% volume shrinkage on cure, the calculated density corresponds very closely with the data of Lewis et al., suggesting that their composites are essentially void-free, as are ours for volume fractions up to 34%. This is consistent with their thermal conductivity results, which compare well with ours and with others in the literature [3].

The second reference for comparison of the density values is Chung and Lin [12], who use two different sizes of BN platelets, 3.6 μm and 10.6 μm, in a matrix of cresol novolac epoxy cured with a phenol novolac hardener and 1-benzyl-2-methylimidazole catalyst. These data are included in Figure 4 as filled green triangles and purple squares, respectively, where it can be seen that they fall close to the calculated dashed line, thus following approximately the trend displayed by our own results.

It should be noted that both Lewis et al. [25] and Chung and Lin [12] compact their epoxy-BN mixtures before curing, using pressures of 1.6 MPa and 5.0 MPa, respectively. Zhu et al. [23] investigate the use of much higher consolidation pressures in a DGEBA epoxy system cured with phenolic aldehyde amine (1.01–1.10 g/cm^3^), and with very high BN contents, well beyond those used here. The reported densities, for BN contents between 74 wt.% and 95 wt.% do not appear to show any systematic variation with either BN content or consolidation pressure, and fall well below the “theoretical” density, which increases from about 1.8 g/cm^3^ to about 2.2 g/cm^3^ over this filler range. Indeed, the reported density of approximately 1.0 g/cm^3^ for composite samples fabricated without consolidation before cure is apparently less than that of the unfilled epoxy system (1.14 g/cm^3^). This would appear to be consistent with the reported values of thermal conductivity for these composites cured without prior consolidation, and hence without significant orientation, which, at less than 4.0 W/mK, are very low for such highly filled systems [3], implying significant void content.

**Figure 5:**
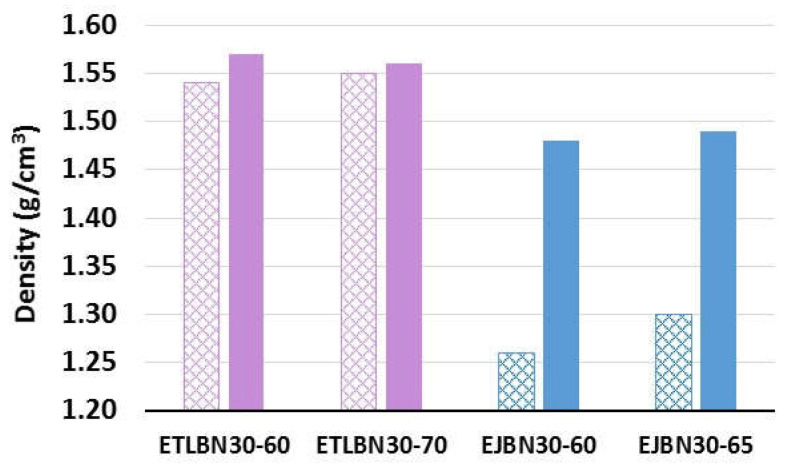
Effect of compression during cure on the density of epoxy–thiol and epoxy–diamine composites with 30 μm BN platelets, as indicated; cross-hatched = ambient pressure, filled = compressed at 175 kPa.

The effect on the density of curing the samples under a pressure of 175 kPa is illustrated in Figure 5 for epoxy–thiol and epoxy–diamine composites filled with 30 μm BN platelets. For both composite systems the filler loadings used were the two highest weight proportions. For the epoxy–thiol system, the compressed samples, denoted ETLBN30-60C and ETLBN30-70C, had filler contents of approximately 34 and 45 vol.%. For the epoxy–diamine system, the compressed samples, denoted EJBN30-60C and EJBN30-65C, had filler contents of approximately 38 and 43 vol.%. 

The difference between the effects of curing under pressure for the two composite systems is remarkable. For the epoxy–thiol composites, the increase in density resulting from curing under this rather low pressure of 175 kPa is small, as can be seen in Figure 5, but can be considered significant when the results for all the composites systems, collected in Table 2, are examined. The values listed in Table 2 show that the density increases systematically, albeit in a modest way, for the epoxy–thiol composite samples with both 60% and 70% of the 30 μm and 180 μm platelets when they are cured under a pressure of 175 kPa.

Strangely, however, the density appears to decrease for the samples filled with 2 μm platelets, though the difference is small and close to experimental uncertainty. This is probably related to the much higher specific surface area, 10 m^2^/g, for these platelets in comparison with that for the larger platelets, 1 m^2^/g, which made it very difficult to fabricate composites with a high filler content of 2 μm platelets. It can be seen, for example, that no data are included in Figure 4 for the 70% content of 2 μm platelets; the texture of the uncured mixture was quite different from that for the other platelet sizes, being somewhat powdery and unable to be compacted.

**Figure 6:**
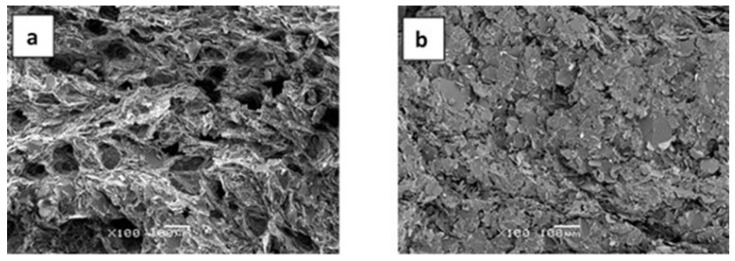
SEM micrographs of epoxy–diamine samples: (**a**) EJBN30-60 and (**b**) EJBN30-60C. Magnification 100×, scale bar 100 μm.

In comparison with the very limited effect of the pressure of 175 kPa on the density of the epoxy–thiol composites illustrated in Figure 5, even this rather low pressure is sufficient to cause a dramatic increase in density for the epoxy–diamine composites. This can be understood from the SEM micrographs of the fracture surfaces, shown in Figure 6, where the voids in the sample prepared at atmospheric pressure (Figure 6a) have largely been eliminated by curing under 175 kPa pressure (Figure 6b). The reason why the problem of voids is not apparent in the epoxy–thiol samples is explained by the much lower viscosity of the epoxy–thiol mixtures.

It is remarkable that the large density changes observed in the epoxy–diamine composites shown in Figure 5 should occur at the pressure of only 175 kPa, which is very low in comparison with many of the pressures used by other workers, as summarised in the Introduction. However, there are other examples of similarly low pressures having a significant effect on the properties of epoxy composites. Zhang et al. [31] hot press their epoxy-BN composites at just 207 kPa (30 psi) and obtain a significant orientation of the BN platelets, with a corresponding enhancement of the thermal conductivity in the in-plane direction. In the present case of the epoxy–diamine composites, we attribute the increase in density to the elimination of voids, which is an important consideration for fibre-reinforced epoxy composite systems. For example, Thomason [40] and Gu et al. [41] show that large reductions in the void content can be achieved using pressures well below 1 MPa; in fact, increasing the pressure from 90 to 270 kPa can reduce the void content by more than a factor of 2 [41].

At the higher pressure of 2 MPa, it can be seen from the results included in Table 2 that there is a significantly greater increase in density for the epoxy–thiol composites fabricated with both 30 μm and 180 μm platelets. In fact, it appears that the densities of these samples, 1.70 g/cm^3^ and 1.69 g/cm^3^, respectively, are greater than the calculated density for 70% epoxy–thiol-BN composites, 1.64 g/cm^3^ (see Figure 4); this value is calculated assuming simple additivity of stoichiometric amounts of epoxy and thiol, with densities of 1.17 g/cm^3^ and 1.28 g/cm^3^, respectively, and the appropriate proportion of BN platelets with a density of 2.1 g/cm^3^, and allowing for a 4% shrinkage on cure. These results for the densities of the epoxy–thiol-BN composites cured at 2 MPa pressure therefore require some comment.

One possibility is related to the effect of pressure on the cure process, and in particular on the density of the cured epoxy. Although there is one report that the density is independent of the cure pressure, in the range up to 1000 MPa [42], the majority of studies suggest that increasing the cure pressure results in a higher density of the cured epoxy [43,44,45,46]. Beloshenko and co-workers [43,44] investigated various epoxy resins and found that increasing pressure during cure results in a higher density of the cured epoxy, attributing this effect to the inhibition of voids and a reduction of the free volume. This latter aspect is equivalent to the effects of densification, discussed elsewhere [22], which requires not only that the cure be made under pressure but also that the pressure be maintained while the sample is cooled from the curing temperature. Hwang and Chang [45] used a plunger-type dilatometer to measure the volume shrinkage during cure of an epoxy molding compound (EMC), and observed an increase in the volume shrinkage with increasing pressure in the range from 2 to 10 MPa. Hopmann et al. [46] used a specially constructed dilatometer to measure the cure shrinkage and found that, for a low viscosity epoxy resin cured with an amine, the shrinkage increased from about 4% to about 7% when the pressure was increased to 2.5 MPa. Accordingly, it is possible that, for our epoxy–thiol system, the combined effects of the increased shrinkage due to curing under pressure and the densification as a consequence of maintaining the pressure during cooling could have increased the density of the cured epoxy-BN composite beyond the anticipated value.

In clear contrast to the increase in the density observed for the epoxy–thiol composite systems with BN particles in the form of platelets discussed above and illustrated in Table 2, there is no significant increase in density for composites fabricated with the 120 μm spherical agglomerates; the reason for this is considered below.

### 3.2. Thermal Conductivity

**Figure 7:**
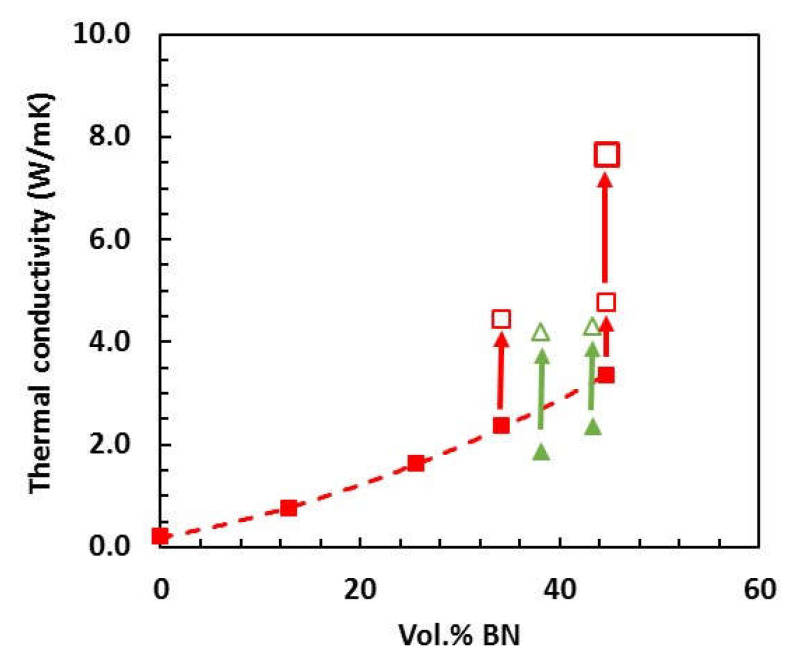
Thermal conductivity as a function of BN content for ETLBN30 samples (red squares) and EJBN30 samples (green triangles): filled symbols, samples cured at ambient pressure [3,4,5,6]; small open symbols, samples cured under 175 kPa pressure; large open symbol, sample cured under 2 MPa pressure.

The effect of curing under pressure on the thermal conductivities of the epoxy–thiol and epoxy–diamine composites with 30 μm BN platelets is shown in Figure 7, the complete set of data being given in Table 2. For comparison, additional results for epoxy–thiol samples cured at ambient pressure and covering a wider range of BN contents (filled symbols) are taken from earlier work [3,4,5,6], and show how the thermal conductivity increases non-linearly with increasing BN content. To achieve higher thermal conductivities than about 3 W/mK in composites prepared at ambient pressure (filled symbols) would require an increased BN content, but the problem is that the uncured mixture becomes such a stiff paste at BN contents greater than about 45 vol.% that it is essentially unworkable. On the other hand, the application of pressure during cure, a feasible fabrication procedure using, for example, an autoclave, results in a large increase in the thermal conductivity for the two BN contents used here, as can be seen in Figure 7. For both the epoxy–thiol-BN and epoxy–diamine-BN composite systems, the use of even the rather low pressure of 175 kPa results in an increase in thermal conductivity by as much as a factor of 2, as indicated by the arrows. For the epoxy–thiol system and a cure pressure of 2 MPa, the increase in thermal conductivity is even more dramatic.

**Figure 8:**
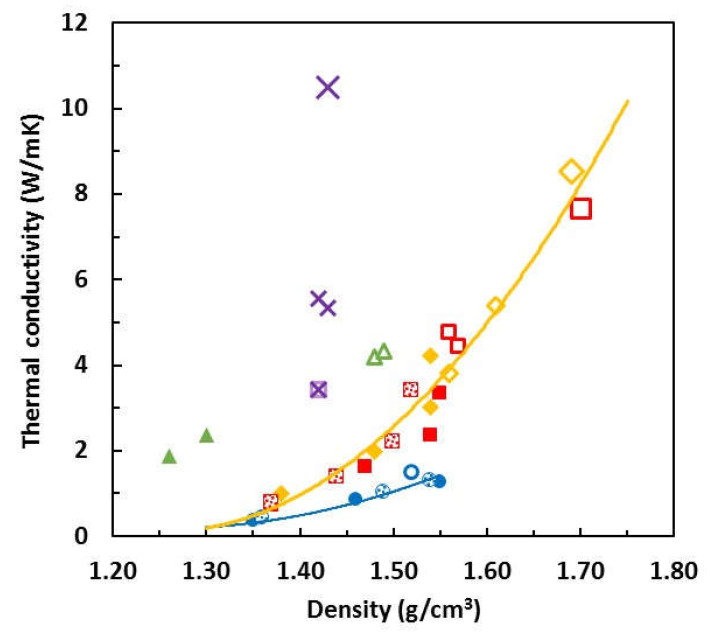
Thermal conductivity as a function of the density for the epoxy-BN composites. Full symbols, ambient pressure cure with LC-80 [3,4,5,6]; shaded symbols, ambient pressure cure with PDU [3,4,5,6]; small open symbols, cured under 175 kPa pressure; large open symbols, cured under 2 MPa pressure. ETLBN2, blue circles; ETLBN30, red squares; ETLBN180, yellow diamonds; EJBN30, green triangles; ETLBN120s (agglomerates), purple crosses.

The preceding observations, which show that both the density and the thermal conductivity increase when the samples are cured under pressure, and more so the higher is the pressure, raise the question of whether or not these two properties are inter-related in respect of the effect of pressure. In Figure 8, the thermal conductivity is plotted as a function of the density for all the epoxy-BN composites studied. Included here also are earlier results for the epoxy–thiol system cured at ambient pressure using either PDU (shaded symbols) or LC-80 (full symbols) as the initiator [3,4,5,6].

There appears to be quite a strong correlation between the thermal conductivity and the density, within certain groups of BN filler types. For the 30 μm and 180 μm platelets, for both of which the specific surface area is 1.0 m^2^/g, an increase in the density as a consequence of applying pressure during cure results in an increase in the thermal conductivity which follows the trend of the increase resulting from higher BN contents, indicated by the full yellow curve. This may be explained on the basis of the thermal conductivity depending on the pathways for heat conduction between the BN particles.

A similar correspondence between thermal conductivity and density was noted by Zhu et al. [23], though the pressure was applied in quite different circumstances, specifically for consolidating and orienting the BN platelets within the samples before curing at ambient pressure. Nevertheless, the thermal conductivity in the in-plane direction increases in an apparently non-linear manner, similar to our results in Figure 8, and reaches a maximum value just greater than 21 W/mK, even though this was not for the highest pressure applied. No report is made for the equivalent relationship for the thermal conductivity in the through-plane direction, which reaches a value of approximately 12 W/mK at the highest BN content, between 90 and 95 wt.%.

These very high values of thermal conductivity obtained by Zhu et al. [23] are a consequence of two particular aspects: the BN platelets are oriented by the application of pressure, and the filler contet is very high. To compare our present results for thermal conductivity with others in the literature, we set aside those, such as Zhu et al., in which significant orientation is introduced by the composite preparation process, and we make use of the compilation of data presented in an earlier review [3]. It is remarkable that our values for samples with 58.0 wt.% BN and prepared under 2 MPa pressure, namely 7.67 W/mK and 8.52 W/mK for composites with 30 μm and 180 μm platelets, respectively, and 10.49 W/mK for composites with 120 μm agglomerates (see Table 2), are higher than any others reported, with one exception. The one exception is Islam et al. [47], who make use of a liquid crystalline epoxy resin cross-linked either with an amine or a cationic initiator. This unfilled resin has an intrinsically higher thermal conductivity than DGEBA, 0.34 ± 0.02 W/mK for amine cure and 0.45 ± 0.03 W/mK for cationic cure, and when filled with 55 wt.% (the closest to the content of our ETLBN*x*-70 samples, 58.0 wt.%) of 30 μm BN platelets gives values of 8.77 W/mK and 10.96 W/mK for amine and cationic cure, respectively. These composite samples are prepared by hot-pressing at 20 MPa, but the authors verify that there is no orientation induced by this procedure. Our values for the epoxy–thiol system at 2 MPa are therefore only slightly less than those of Islam et al. at 20 MPa, which represent the highest values reported for isotropic epoxy-BN composites. It is possible that the epoxy–thiol values could be increased further by pressures higher than 2 MPa.

For comparison, we note the following studies in which thermal conductivities are reported with values which most closely approach our own. Xu and Chung [7] report a thermal conductivity of 10.31 W/mK for samples with 57 vol.% (~70 wt.%) of silane treated BN particles, 5–11 μm in size, prepared at 10.5 MPa pressure, but at 44 vol.% (~58 wt.%) the corresponding values is only 4.84 W/mK. Chung and Lin [12] prepared composites with 10.6 μm silane treated BN platelets, cured in a vacuum oven after pressing a mold at 5 MPa, and found a strong dependence of thermal conductivity on BN content, which passed through a maximum between 50 and 60 vol.%. Their values of 6.75 W/mK at 50 vol.% (~65 wt.%) and 7.42 W/mK at 60 vol.% (~74 wt.%) are less than our values at a lower filler content of 58 wt.%. Lastly, Kargar et al. [48] reported a value of 5.50 W/mK for their highest content of 45 vol.% (~59 wt.%) of 3–8 μm BN platelets, which, though higher than many other values reported in the literature [3], is still significantly less than our values at a similar filler content. 

The apparent correlation between thermal conductivity and density demonstrated by Figure 8 can be explained as follows. Both increased BN content and the application of pressure during cure increase the thermal conductivity and improve the heat transfer. In the former case, increasing the BN content increases the number of heat conduction pathways and simultaneously increases the density, as a consequence of the higher density of the BN particles in comparison with the epoxy matrix. In the latter case, the application of pressure during cure improves the matrix–filler interface as a consequence of the increased density. A similar trend is seen for the 2 μm platelets, indicated by the blue curve, though it is displaced to much lower thermal conductivities. This results from the much higher specific surface area, 10 m^2^/g, for these BN particles, which creates a much larger interfacial area between matrix and filler.

**Figure 9:**
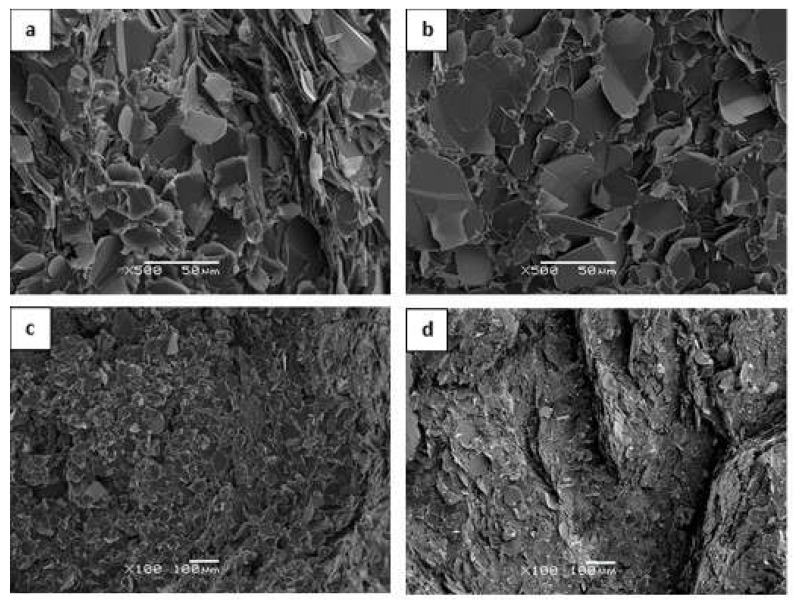

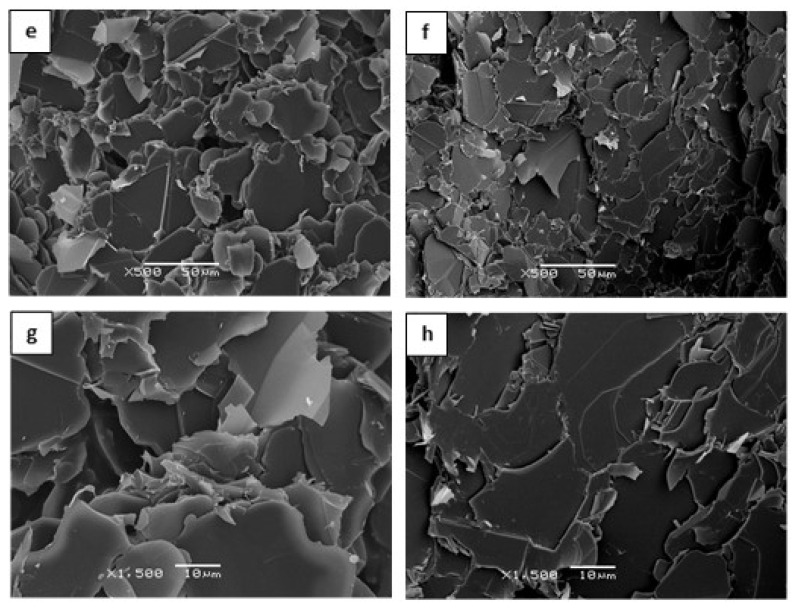
SEM micrographs of fracture surfaces of epoxy–thiol composites filled with BN platelets: (**a**) ETLBN30-60 and (**b**) ETLBN30-60C, with magnification 500×; (**c**,**e**,**g**) ETLBN30-70 and (**d**,**f**,**h**) ETLBN30-70C2 with magnifications 100×, 500×, 1500×, respectively. Scale bars are indicated on each micrograph.

The effect of applying pressure during cure on the pathways for heat conduction is illustrated in Figure 9 by the SEM micrographs of the epoxy–thiol composites fabricated with 30 μm platelets. Samples ETLBN30-60, cured at ambient pressure, and ETLBN30-60C, cured at 175 kPa pressure, are shown in Figure 9a,b; samples ETLBN30-70, cured at ambient pressure, and ETLBN30-70C2, cured at 2 MPa pressure, are shown in Figure 9c–h at different magnifications.

In comparison with the fracture surfaces of the epoxy–diamine composites, shown above in Figure 6 and which demonstrate that for the epoxy–diamine system the increase in thermal conductivity results principally from a reduction in the void content on the application of pressure during cure, the SEM micrographs for the epoxy–thiol system in Figure 9 show that the void content is not an issue here: there are no voids visible in these micrographs, neither in the samples cured at ambient pressure nor in those cured at 2 MPa. Here the comparison is made between the 60% composites fabricated without and with 175 kPa pressure, Figure 9a,b, respectively, and between the 70% composites fabricated without and with 2 MPa pressure, Figure 9c,e,g on the one hand and Figure 9d,f,h on the other.

It can be seen that for the samples cured under pressure there is much better contact between the BN particles and an improved matrix–filler interface. The improvement noted between Figure 9c,e,g on the one hand and Figure 9d,f,h on the other, as a consequence of curing at 2 MPa, is significantly better than the improvement between Figure 9a,b, for cure at 175 kPa. Similar observations were made for the ETLBN180 composites. This supports our view that the effect of pressure in the epoxy–thiol system filled with BN platelets is to bring the BN particles into closer and better contact, and hence increase the thermal conductivity.

On the other hand, the epoxy–thiol composites with 120 μm agglomerates display a quite different relationship between thermal conductivity and density, in which the thermal conductivity increases dramatically without a significant increase in the density. This is very clearly seen in Figure 8 where the data for the composites fabricated with agglomerates are shown as the open purple crosses. The value of over 10 W/mK for the composite filled with approximately 45 vol.% of 120 μm spherical agglomerates (about 58 wt.%) is higher than any other value reported in the literature for the same filler content [3], except for those for which elaborate fabrication techniques are required, which are not usually practical. This suggests that the increased thermal conductivity for these composites with BN agglomerates occurs for reasons other than simply having an improved matrix–filler interface, and this can indeed be shown by reference to the fracture surfaces observed by SEM.

**Figure 10:**
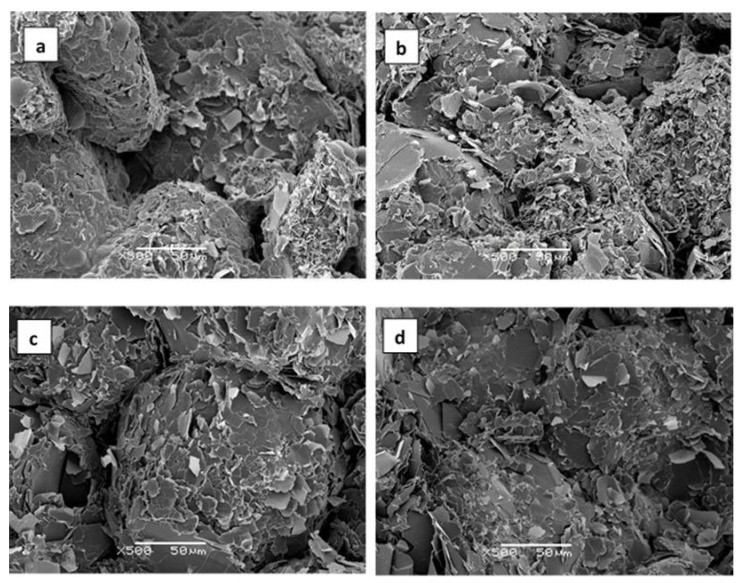
SEM micrographs of fracture surfaces of epoxy–thiol composites filled with BN agglomerates: (**a**) ETLBN120s-60, (**b**) ETLBN120s-60C, (**c**) ETLBN120s-70, and (**d**) ETLBN120s-70C2. Magnification 500×, scale bar 50 μm.

The SEM micrographs for the epoxy–thiol composites filled with BN agglomerates are shown in Figure 10, both for the ETLBN120s-60 composites prepared at ambient pressure (Figure 10a) and with 175 kPa pressure (Figure 10b), and for the ETLBN120s-70 composites prepared at ambient pressure (Figure 10c) and at 2 MPa pressure (Figure 10d).

In Figure 10a the individual agglomerates can clearly be seen, essentially with their original approximately spherical form as shown in Figure 1, and with a certain, but limited, area of contact with their neighbouring particles. In Figure 10b, though, these agglomerates can be seen to have been deformed by the pressure, such that the area of contact between neighbouring particles is now considerably greater. The same observations can be made for the ETLBN120s-70 composites in Figure 10c,d for the samples formed without pressure and with 2 MPa pressure, respectively. It is this deformation of the agglomerates and the consequent increase in thermal contact between the particles which leads to the dramatically increased thermal conductivity seen in Figure 8. Since particles in the form of agglomerates are the most efficient for increasing the thermal conductivity of epoxy-BN composites with a given filler content [3,4,5], the application of pressure would therefore appear to be an excellent way in which to enhance the thermal conductivity.

## 4. Conclusions

The application of pressure during the cure of several different epoxy-BN composite systems has been shown to increase both the density and the thermal conductivity of these composite materials. In most cases there is a correlation between the density and the thermal conductivity, but the mechanisms for enhancing the thermal conductivity depend on the particular epoxy-BN system. For the epoxy–diamine system, the principal effect of applying pressure is to reduce the void content, which leads to a large increase in the thermal conductivity. On the other hand, for the epoxy–thiol system in which the BN filler particles are in the form of platelets, the enhancement in thermal conductivity results from an improvement in the matrix–filler interface, and the closer proximity of the particles. In contrast, for the epoxy–thiol system filled with BN particles in the form of agglomerates, the effect of pressure is to deform the agglomerates, leading to a greatly increased area of contact between the particles and a correspondingly dramatic increase in the thermal conductivity. The use of pressure during cure, particularly for epoxy-BN composites filled with particles in the form of agglomerates, is therefore considered to be an excellent procedure for achieving high thermal conductivities in these systems.

## Figures and Tables

**Table 1:** Compositions by weight of epoxy–thiol samples, ETLBN, with BN particles (particle size, *x* = 2, 30, 120 s, 180 μm) and epoxy–diamine samples, EJBN, with BN particles (*x* = 30). Note that ETLBN2-70 samples could not be prepared. The weight per cent of BN particles is converted into volume per cent in the final column.

Sample	Epoxy	BN	Thiol	LC-80	Diamine	BN vol.%
ETL	100	0	66.7	2.0	-	0
ETLBN*x*-30	70	30	46.7	1.4	-	12.9
ETLBN*x*-50	50	50	33.4	1.0	-	25.7
ETLBN*x*-60	40	60	26.7	0.8	-	34.2
ETLBN*x*-70	30	70	20.0	0.6	-	44.7
EJ	100	0	-	-	33.3	0
EJBN*x*-60	40	60	-	-	13.3	38.1
EJBN*x*-65	35	65	-	-	11.7	43.3

**Table 2:** Densities and thermal conductivities (TC) of composites, ETLBN*x*-*y* and EJBN*x*-*y*, cured at ambient pressure, *P*_amb_, and those cured with 175 kPa and 2 MPa pressure, where *x* refers to the particle size and *y* refers to the filler content. Samples cured under 175 kPa pressure are labelled ETLBN*x*-*y*C and those cured under 2 MPa pressure are labelled ETLBN*x*-*y*C2.

Sample	*P*_amb_	Density (g/cm^3^) 175 kPa	2 MPa	*P*_amb_	TC (W/mK) 175 kPa	2 MPa
ETLBN2-60	1.55	1.52	-	1.28	1.49	-
ETLBN30-60	1.54	1.57	-	2.36	4.44	-
ETLBN30-70	1.55	1.56	1.70	3.34	4.77	7.67
ETLBN180-60	1.54	1.54	-	3.02	3.80	-
ETLBN180-70	1.54	1.61	1.69	4.22	5.38	8.52
ETLBN120s-60	1.42	1.43	-	3.42	5.34	-
ETLBN120s-70	-	1.42	1.43	-	5.55	10.49
EJBN30-60	1.26	1.48	-	1.87	4.19	-
EJBN30-65	1.30	1.49	-	2.37	4.32	-

## Data Availability

The data presented in this study are available on request from the corresponding author.
